# Biological Activities of Glass Ionomer Cement Supplemented with Fortilin on Human Dental Pulp Stem Cells

**DOI:** 10.3390/jfb13030132

**Published:** 2022-08-28

**Authors:** Prawichaya Sangsuwan, Sissada Tannukit, Wilaiwan Chotigeat, Ureporn Kedjarune-Leggat

**Affiliations:** 1 Molecular Biology and Bioinformatics Program, Faculty of Science, Biological Science Division, Prince of Songkla University, Hat Yai 90110, Thailand; 2 Department of Oral Biology and Occlusion, Faculty of Dentistry, Prince of Songkla University, Hat Yai 90110, Thailand; 3 Cell Biology and Biomaterial Research Unit, Faculty of Dentistry, Prince of Songkla University, Hat Yai 90110, Thailand

**Keywords:** fortilin, human dental pulp stem cells, glass ionomer cement

## Abstract

This study aimed to determine the most suitable recombinant fortilin and evaluate the biological activities of glass ionomer cement (GIC) incorporated with fortilin on human dental pulp stem cells (hDPSCs). Full-length and three fragments of *Penaeus merguiensis* fortilin were cloned and examined for their proliferative and cytoprotective effects on hDPSCs by MTT (3-(4, 5-dimethylthiazol-2-yl)-2, 5-diphenyltetrazolium bromide) assay. Human DPSCs were cultured with GIC supplemented with fortilin, tricalcium phosphate, or a combination of tricalcium phosphate and fortilin, designated as GIC + FL, GIC + TCP, and GIC + TCP + FL, respectively (*n* = 4 for each group). At given time points, hDPSCs were harvested and analyzed by MTT, quantitative reverse transcription polymerase chain reaction, alkaline phosphatase activity, and Alizarin Red assays. The full-length fortilin promoted cell proliferation and significantly increased cell survival. This protein was subsequently added into the GIC along with tricalcium phosphate to investigate the biological activities. All experimental groups showed reduced cell viability after treatment with modified GICs on days 1 and 3. The GIC + TCP + FL group significantly promoted odontoblastic differentiation at particular time points. In addition, alkaline phosphatase activity and calcium phosphate deposit were markedly increased in the GIC + TCP + FL group. Among all experimental groups, the GIC incorporated with fortilin and tricalcium phosphate demonstrated the best results on odontogenic differentiation and mineral deposition in hDPSCs.

## 1. Introduction

Glass ionomer cement (GIC) is a dental material that has been used in clinical dentistry for a wide range of applications such as filling, lining, and luting purposes [1]. Originally, GIC was composed of fluoroaluminosilicate glass and polyalkenoic acids in aqueous solution, which contain polyacrylic acid as the main component. Conventional GIC possesses several advantages including chemical bond formation with the tooth structure and fluoride release [1]. In an effort to improve the properties of GICs, many studies have aimed at the development of GIC-based materials with better biological properties. Modified materials incorporated with biological molecules, such as growth factors, have gained increased attention in materials research [2,3]. These biological molecules have been reported to modulate cell behavior such as cell migration, cell proliferation, and cell differentiation [2,4]. Induction of dentin regeneration is one of the key goals for treatment of exposed dental pulp with capping material. Improvement of pulp-capping materials with regenerative capacity is challenging since the dentin–pulp complex has limited capacity to remodel.

Fortilin, also referred to as translationally controlled tumor protein (TCTP), is a histamine releasing factor (HRF) that has been extensively studied for its anti-apoptotic function [5,6]. Fortilin was first identified as a protein with an expression that was controlled at the translational level in mouse tumor cells [7]. Subsequently, fortilin has been widely studied and has demonstrated multiple functions in cell growth, survival, and apoptosis [5,8]. In addition to its intracellular functions, fortilin can act as a cytokine by stimulating histamine release [9]. Previous studies have shown increased fortilin expression following exposure to various types of stimuli such as heat, where it offers protection to cells from heat shock [10,11]. Fortilin can protect cells from heat shock and is markedly upregulated in many cell types after exposure to thermal stimuli [11]. Furthermore, fortilin has been shown to promote cell survival from a noxious chemical agent, 2-hydroxyethyl methacrylate (HEMA) [12,13]. Recombinant fortilin from *Penaeus merguiensis* (*Pmer*-fortilin) was shown to have an anti-apoptotic function in HEMA-treated human dental pulp cells [12,13]. A previous study showed that *Pmer*-fortilin shares approximately 44% similarity with its human counterpart [14]. Fortilin was reported to have anti-apoptotic activity across different species, and its respective mechanisms were demonstrated through several pathways [15]. Recently, it was shown that fortilin can promote proliferation and differentiation of human osteoblasts [16]. In addition, resin-modified GIC supplemented with recombinant fortilin was shown to have reduced cytotoxicity [16].

During the restorative procedure, pulpal irritation is triggered by several factors such as heat during cavity preparation and toxins from dental materials. Depending on the severity of stimuli, the pulpal response may result in mild inflammation, irreversible pulpitis, or apoptosis [17]. Due to its anti-apoptotic function, fortilin bears promise in its potential application for the development of bioactive dental materials. It is also worthwhile to examine the biological activities of different fragments of full-length fortilin. The aims of this study were to determine the most suitable recombinant fortilin and investigate the biological properties of GIC modified with fortilin on human dental pulp stem cells. The null hypothesis was that there was no difference in biological properties among GIC and three different formulations of modified GICs.

## 2. Materials and Methods

### 2.1. Plasmid Construction and Expression of Recombinant Fortilins

*Pmer*-fortilin was obtained by polymerase chain reaction (PCR) and ligated in frame to bacterial expression vector. The full-length sequence was divided into three fragments according to the conserved regions, which are mainly located at the N-terminus and sparsely scattered throughout the rest of the sequence. To create truncated fragments encoding residue 1–60, 61–100, and 101–168, full-length construct was used as a template and amplified by the appropriate primer sets. The PCR products were cloned into *pGEM-T Easy* (Promega) vector and the positive clones were verified by DNA sequencing. The full-length fortilin and truncated fragments were then cloned into *pGEX-4T-1* vector (Amersham, Thailand) and transformed into *Escherichia coli* (*E. coli*) strain BL21. Subsequently, the protein was expressed and purified following the method previously described [16]. The proteins were subsequently resolved via SDS-PAGE. Based on the amino acid sequence, the different fragments of recombinant fortilin were designated as FL1-60, FL61-100, and FL101-168 accordingly.

### 2.2. Cultures of Human Dental Pulp Stem Cells (hDPSCs)

Primary dental pulp cells were collected from human third molars of three adults aged between 18 and 25 years. All subjects signed consent forms following the study protocol approved by the Research Ethics Committee (code no. EC6211-047). The pulp tissue was separated and minced to an approximate size of 1–2 mm^2^. The tissue was then digested in a pre-warmed mixture of collagenase and dispase (Gibco, Life Technologies Corporation, Carlsbad, CA, USA) at 37 °C for 45 min. After incubation, the cells were spun down and the cell pellets were collected. The cells were resuspended in alpha modified Eagle’s medium (α-MEM) containing 20% fetal bovine serum (FBS), 100 µM L-ascorbic acid 2-phosphate, 100 µM L-glutamate, 100 units/mL penicillin, and 100 µg/mL amphotericin, and were cultured at 37 °C in a 5% CO_2_ incubator. The cells were subcultured after reaching confluency and were defined as the first passage. The stem cell markers were examined using Human MSC Analysis kit (Beckman Coulter, Villepinte, France). Cells were analyzed by Cytoflex flow cytometer (Beckman Coulter, Villepinte, France). The self-renewal capacity of hDPSCs was determined by the colony-forming unit fibroblast (CFU-F) assay. The hDPSCs were seeded at a density of 100 cells/well and incubated for 10 days. Cells were then fixed and stained with 0.4% crystal violet. To detect the differentiation potential, the cells were cultured in osteogenic and adipogenic differentiation media for 14 days. Subsequently, the cells were stained with Alizarin Red S, and Oil red O to detect mineralization nodules and lipid droplets, respectively.

### 2.3. Cell Viability Testing

The effect of recombinant fortilin on cell viability of hDPSCs was investigated by MTT assay. Human DPSCs were seeded at 5 × 10^3^ cells/well in 96-well plate. The cultured cells were exposed to various concentrations of recombinant fortilin for 24 and 72 h. The viable cells were determined by MTT assay. To examine the protective effect of recombinant fortilin, hDPSCs were seeded at 1 × 10^4^ cells/well in 96 well-plate and cultured for 24 h. The cultured cells were then treated with a combination of 8 mM HEMA (Sigma-Aldrich, St. Louis, MO, USA) and various concentrations of recombinant fortilin. After 24 h incubation, the culture medium was refreshed and the cells were incubated for 48 h. The cell viability was determined by MTT assay. The formazan product was measured at 570 nm. The optical density (OD) values of the experimental groups were expressed as percentage of viable cells.

### 2.4. Histamine Releasing Assay

The peripheral blood mononuclear cells (PBMCs) were separated from whole blood of three subjects by Ficoll-Hypaque density gradient. PBMCs were suspended in phosphate-buffered saline (PBS) containing 1 mM Ca^2+^ and 1 mM Mg^2+^ and 0.03% (*w*/*v*) bovine serum albumin (BSA). The cells were treated with various concentrations of full-length fortilin at 1, 10, and 100 µg/mL, and calcium ionophore A23187 (10 µM) as a positive control. After incubation for 30 min, the cells were centrifuged at 1300× *g* for 10 min at 4 °C, and the supernatant was collected. Histamine released into the supernatant was measured by the fluorometric technique, using o-phthalaldehyde (OPT) (Sigma-Aldrich, St. Louis, MO, USA). Briefly, the supernatant was extracted by 0.4 M perchloric acid and centrifuged at 2000× *g* for 15 min. The collected supernatant (5 mL) was then mixed with butanol (12.5 mL) and 2.5 M NaOH (1 mL) and incubated for 10 min. Subsequently, 10 mL of the butanol phase was transferred to a mixture of heptane and 0.1 M HCl (12.5 mL) and then vortexed. The HCl phase (2 mL) was then mixed with 400 µL of 10% OPT and 800 µL of 0.05 M NaOH, and incubated for 10 min. To terminate the reaction, 800 µL of 1 M H_3_PO_4_ was added and the fluorescent intensity was measured using a spectrofluorometer at 450 nm. The results were reported as the level of histamine released (mg/mL) from an individual subject.

### 2.5. Specimens Preparation

The specimens were prepared using the powder/liquid ratio recommended by the manufacturer. Under aseptic conditions, the powder and the liquid were mixed using sterile material in a laminar fume hood. Recombinant fortilin (1 µg/specimen) was added into the GIC (Ketac-Molar Easymix; 3M ESPE, MN, USA) during mixing. After mixing, the material was packed into a sterile Teflon mold, which is 5 mm in diameter and 1 mm in thickness. The GIC discs were allowed to set and stored at 37 °C for 1 h prior to use for the experiment. There was 1 control group designated as GIC and 3 groups of modified GICs designated as GIC + TCP, GIC + FL, and GIC + TCP + FL, details of which are described in Table 1.

### 2.6. Scanning Electron Microscopy (SEM)

The GIC discs were dehydrated in a graded series of ethanol and subsequently examined with SEM Quanta 400 (Thermo Fisher Scientific, Waltham, MA, USA). Images were captured at 1000× magnification.

### 2.7. Cytotoxicity of Modified GICs on hDPSCs

The hDPSCs were seeded at 7 × 10^3^ cells on each GIC disc. The cells were cultured for 1, 3, and 5 days. On the day of assessment, the viable cells were determined by MTT assay. The formazan product was measured at 570 nm.

### 2.8. Quantitative Reverse Transcription Polymerase Chain Reaction (qRT-PCR)

The hDPSCs were seeded at 1 × 10^5^ cells on each GIC disc and cultured for 7, 14, and 21 days. Total RNA was extracted and purified using PureLink™ RNA Mini Kit (Invitrogen, USA). The cDNA was generated by reverse transcription using the SuperScript III First-Strand Synthesis System (Invitrogen, USA). PCR was performed using the SensiFAST™ SYBR**^®^** No-ROX Kit (Bioline, UK) on the Light Cycler System (Roche Diagnostics, Mannheim, Germany). The primers for each target genes are listed in Table 2. The PCR condition was set as follows: one cycle of denaturation at 95 °C for 10 min, repeated cycles of denaturation at 95 °C for 30 s, annealing at 58 °C for 30 s, and elongation at 72 °C for 45 s. GAPDH was used as a reference gene for each target gene. The relative quantifications were calculated using the 2^−ΔΔCT^ method.

### 2.9. Alkaline Phosphatase (ALP) Assay

The hDPSCs were seeded at 1 × 10^4^ cells on each GIC disc. At given time points, cell lysates were then harvested with lysis buffer (1% Triton-X100 in PBS pH 7.4). The ALP activity was determined by adding 4-nitriphenyl phosphate in 0.2 M of amino-2-methyl-l-propanol and incubated for 30 min. To terminate the reaction, 0.1 M NaOH was added and the absorbance was measured at 405 nm. Total protein concentration was measured using the BCA protein assay kit (Thermo Fisher Scientific, Waltham, MA, USA). The activity was calculated using the formula; the enzyme activity = µmole/min/µg of the protein.

### 2.10. Alizarin Red S (ARS) Assay

The hDPSCs were seeded at 1 × 10^4^ cells on each GIC disc. After 24 h the medium was replaced with the osteogenic medium consisting of α-MEM, 10% heat-inactivated FBS, 10 mM beta-glycerophosphate, 100 unit/mL penicillin, 100 mg/mL amphotericin B, 0.05 mM L-ascorbic acid 2-phosphate, and 100 mM dexamethasone. The cells were cultured for 7, 14 and 21 days. On the day of assessment, cells were fixed and stained with 40 mM of Alizarin Red S solution. Mineral stains were dissolved in cetylpyridinium chloride (CPC) (Sigma-Aldrich, St. Louis, MO, USA) and the absorbance was measured at 550 nm.

### 2.11. Statistical Analysis

The data were examined for normal distribution using the Shapiro–Wilk test. All data sets were normally distributed and presented as mean ± standard deviation (SD). Comparisons of the results among groups at particular time points were analyzed by one-way analysis of variance (ANOVA) and Tukey’s post hoc test. Statistical significance was set at *p* < 0.05.

## 3. Results

### 3.1. Characterization of Mesenchymal Stem Cells

**Figure 1:**
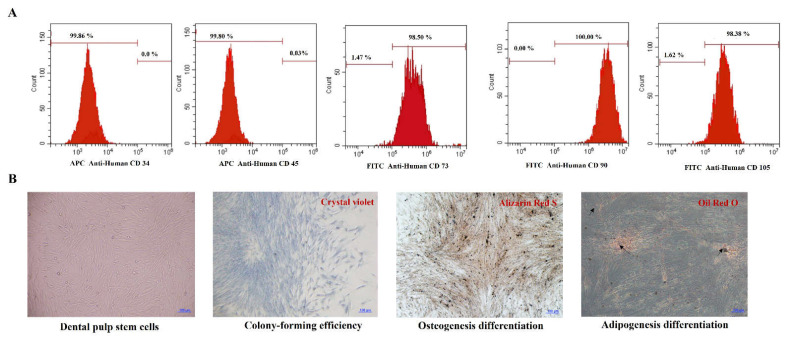
(**A**) Flow cytometry shows positive markers of mesenchymal stem cells; (**B**) Differentiation of stem cells isolated from dental pulp tissue. Cultured cells were stained with Alizarin Red staining and Oil Red O. Scale bars = 100 µm.

Flow cytometry showed that the isolated cells were positive for mesenchymal stem cells markers, CD73, CD90, and CD105, and negative for hematopoietic markers, CD34 and CD45 (Figure 1A). Cells were able to form colonies from the low seeding density, indicating their self-renewal capacity. The cells culturing for 14 days in osteogenic induction medium displayed condensed nodules of calcium stained with Alizarin Red that were sparsely scattered throughout the adherent layer. After 14-day culture in adipogenesis induction medium, lipid droplets in the cytoplasm of the cells stained by Oil Red O were revealed (Figure 1B).

### 3.2. Effect of Full-Length and Fragments of Fortilin on hDPSCs

**Figure 2:**
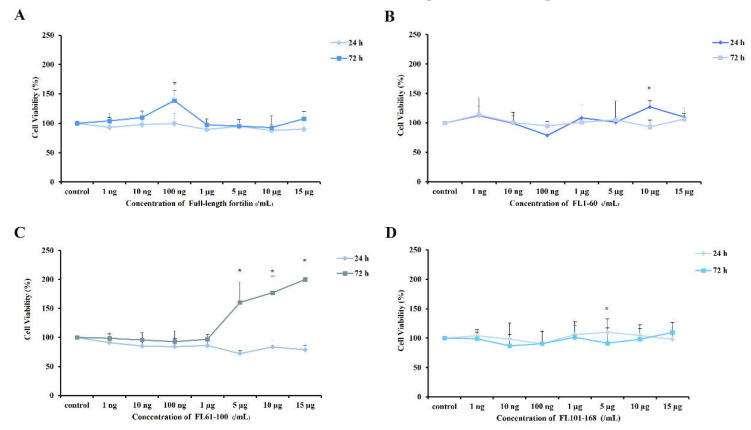
Cell viability after treatment with recombinant fortilins. Cells were treated with various concentrations of fortilin and analyzed by MTT assay. (**A**) The percentage of viable cells treated with full-length fortilin; (**B**) the percentage of viable cells treated with FL1-60; (**C**) the percentage of viable cells treated with FL61-100; (**D**) the percentage of viable cells treated with FL101-168. Data were expressed as means ± standard deviation (*n* = 4). * represents *p* < 0.05 compared to untreated cells (one-way ANOVA with Tukey’s multiple comparison test).

The percentages of cell viability were evaluated after cells were cultured with full-length or fragments of fortilin. At concentrations ranging from 1 ng/mL–15 µg/mL, recombinant fortilin was not cytotoxic to the cells after treatment for 24 and 72 h (Figure 2A–D). Furthermore, fortilins at given concentrations promoted cell proliferation (Figure 2A,C). Full-length fortilin at a concentration of 100 ng/mL significantly enhanced cell viability at 72 h of treatment (*p* < 0.05) (Figure 2A), whereas FL61-100 at concentrations ranging from 5–15 µg/mL promoted cell proliferation after treatment for 72 h in a dose-dependent manner (Figure 2C). Full-length fortilin at concentration of 10 ng/mL was used for further experiments since this was the minimal concentration that promoted cell proliferation.

### 3.3. The Protective Effect of Fortilin on HEMA-Treated hDPSCs

**Figure 3:**
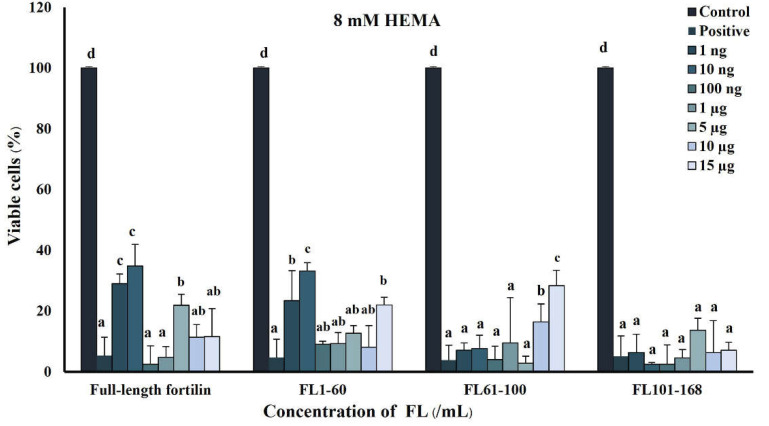
The anticytotoxicity effect of recombinant fortilin and truncated fragments on HEMA-induced cytotoxicity in hDPSCs. Data were expressed as means ± standard deviation (*n* = 4). Different letters represent significance at *p* < 0.01 (one-way ANOVA with Tukey’s multiple comparison test).

The protective effect of full-length and truncated fragments of fortilin against HEMA-induced cytotoxicity was examined by MTT assay (Figure 3). HEMA markedly decreased cell viability, indicating its high toxicity to hDPSCs. Full-length fortilin and FL1-60 at the concentrations of 1 and 10 ng/mL significantly increased cell viability by more than threefold above that of the HEMA-treated cells (*p* < 0.01), whereas FL61-100 showed a comparable effect at a much higher concentration (15 µg/mL). Full-length fortilin at the concentration of 10 ng/mL was used for further experiments since this was the minimal concentration where fortilin promoted cell proliferation and exerted a cytoprotective effect against HEMA.

### 3.4. Histamine Release

**Figure 4:**
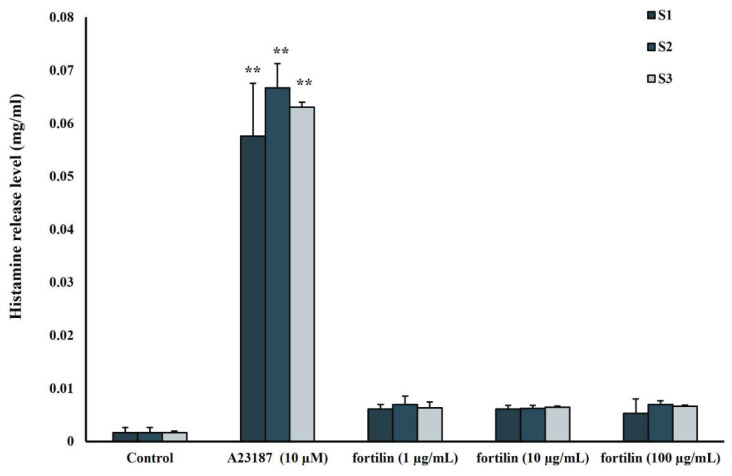
The histamine release level. PBMCs from three subjects designated as S1, S2, and S3, were incubated with 1, 10 and 100 µg/mL of full-length fortilin and calcium ionophore A23187 (10 µM) as a positive control. Data were expressed as means ± standard deviation (*n* = 3). ** represents *p* < 0.01 (one-way ANOVA with Tukey’s multiple comparison test).

To determine whether full-length fortilin can stimulate histamine release, the PBMCs were treated with various concentrations of full-length fortilin and histamine release was examined by fluorometric assay. PBMCs treated with full-length fortilin showed slight induction of histamine release in all subjects (Figure 4).

### 3.5. Morphology of GICs

**Figure 5:**
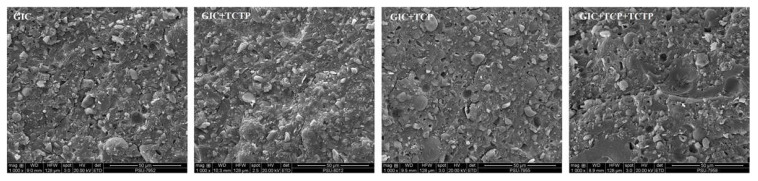
Representative SEM micrographs of GICs at 1000× magnification.

SEM micrographs of GIC specimens are shown in Figure 5. The internal surface of GICs appeared heterogeneous with microcracks visible in all groups. Small voids and pores were observed in the GIC + TCP and GIC + TCP + FL groups.

### 3.6. Cytotoxicity of Modified GICs on hDPSCs

**Figure 6:**
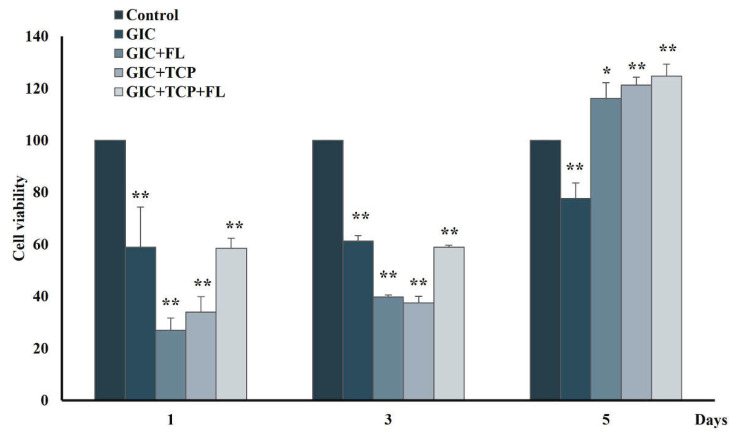
Cytotoxicity of modified GICs on hDPSCs. Cells were incubated with modified GICs for 1, 3 and 5 days, and investigated by MTT assay. Data were expressed as means ± standard deviation (*n* = 4). * and ** represent *p* < 0.05 and *p* < 0.01, respectively (one-way ANOVA with Tukey’s multiple comparison test).

All groups showed reduced cell viability after being treated with GIC specimens for 1 and 3 days. However, the cell viability in all modified GICs was significantly higher than control on day 5 (Figure 6).

### 3.7. Expression of Odontogenic Differentiation Markers in hDPSCs after Cultured with Modified GICs

**Figure 7:**
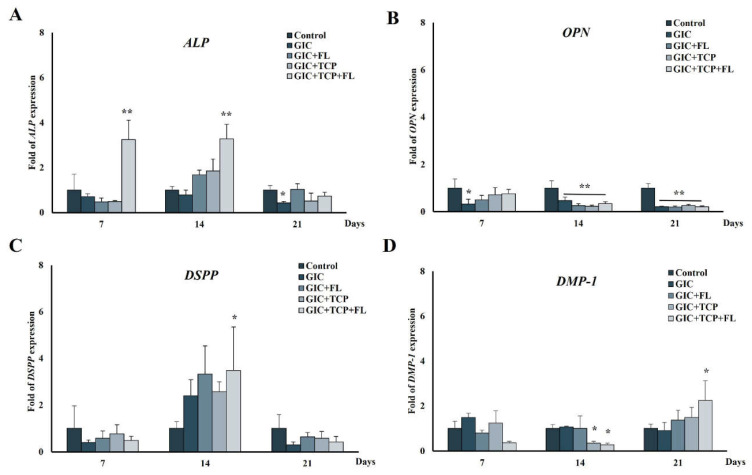
Relative gene expression in hDPSCs cultured on GIC specimens for 7, 14 and 21 days. (**A**) alkaline phosphatase (ALP); (**B**) osteopontin (OPN); (**C**) dentin sialophosphoprotein (DSPP) and (**D**) dentin matrix protein 1 (DMP-1). Data were expressed as means ± standard deviation (*n* = 4). * and ** represent *p* < 0.05 and *p* < 0.01, respectively (one-way ANOVA with Tukey’s multiple comparison test).

To investigate the effect of modified GICs on the differentiation of hDPSCs, the expression of odontogenic differentiation markers was examined by qRT-PCR. In the GIC + TCP + FL group, the mRNA expression of ALP was significantly up-regulated on days 7 and 14 by more than twofold (*p* < 0.01) (Figure 7A), whereas the DSPP and DMP-1 expressions were up-regulated on day 14 and day 21 (*p* < 0.05), respectively (Figure 7C,D). The OPN expression showed significantly decreased level on days 14 and 21 in all groups (Figure 7B).

### 3.8. Effect of Modified GICs on Differentiation of hDPSCs

**Figure 8:**
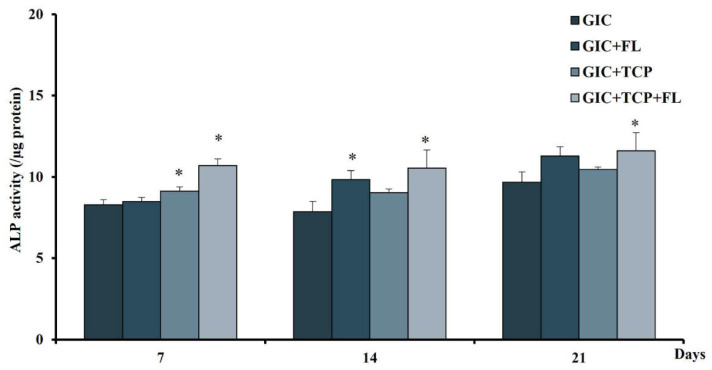
Effect of modified GICs on cell differentiation of hDPSCs. ALP activity was used to evaluate differentiation of hDPSCs on days 7, 14, and 21. Data were expressed as means ± standard deviation (*n* = 4). * represents *p* < 0.05 (one-way ANOVA with Tukey’s multiple comparison test).

To evaluate the effect of modified GICs on cell differentiation, the ALP activity was measured after culturing hDPSCs on the GIC specimens for 7, 14 and 21 days. Alkaline phosphatase activity significantly increased in the GIC + TCP + FL group at all time points investigated, whereas the GIC + TCP and GIC + FL groups showed increased levels of ALP activity at days 7 and 14, respectively (*p* < 0.05) (Figure 8).

### 3.9. Effect of Modified GICs on Mineralization of hDPSCs

**Figure 9:**
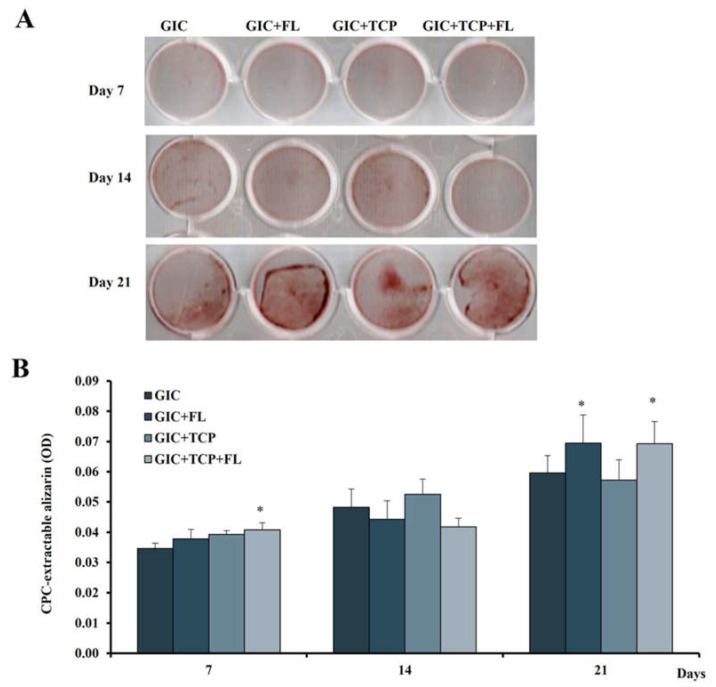
Effect of modified GICs on mineralization of hDPSCs (**A**) representative photographs of Alizarin Red staining of hDPSCs cultured with various GICs for 7, 14 and 21 days; (**B**) optical density of CPC-extracted Alizarin Red. Data were expressed as means ± standard deviation (*n* = 4). * represents *p* < 0.05 (one-way ANOVA with Tukey’s multiple comparison test).

Calcium deposition in hDPSCs cultured with modified GICs for 7, 14 and 21 days was investigated by ARS assay (Figure 9A). All modified GICs showed no difference in calcium deposition on day 14, whereas the GIC+ FL and GIC + TCP + FL groups significantly enhanced the mineralization on day 21 (*p* < 0.05) (Figure 9B).

## 4. Discussion

A number of research studies have been performed on the modification of conventional GICs in order to improve specific biological functionality [18,19,20]. Several approaches including incorporation of bioactive molecules in the materials were developed to promote biological properties such as pulp healing and tissue regeneration [21]. This study intended to develop a bioactive GIC specifically used as a lining cement in deep cavities. In the present study, the most suitable recombinant fortilin was determined and added into the GIC along with TCP to investigate the biological properties. Fortilin was shown to be involved in cell growth, cell cycle progression, apoptosis, and cytoprotection from various stress conditions [5,7,10,22]. The results from the MTT assay indicated that full-length fortilin at concentrations of 10 and 100 ng/mL promoted cell proliferation at 72 h. Although FL61-100 at concentrations of 5–15 µg/mL exhibited a higher proliferative effect than full-length fortilin, this fragment may not be appropriate for further use due to its high concentration and overly proliferative effect that could be detrimental to cells. In addition, the full-length fortilin did not trigger histamine release from PBMCs. Based on the amino acid sequence alignment, *Pmer*-fortilin only shares approximately 44% similarity with its human counterpart [14]. Therefore, *Pmer*-fortilin may not contain the domain responsible for stimulation of histamine release and is unlikely to activate an immune reaction.

In terms of cytoprotective effects, full-length fortilin and FL1-60 at given concentrations appeared to promote cell survival in HEMA-induced apoptosis. These findings indicate that the sequence involved in the anti-apoptotic effect is located in the N-terminal region of *Pmer*-fortilin. This is in accordance with a previous study that reported a BH3-like domain residing at the N-terminal part of human fortilin [23]. This putative BH3 domain of fortilin interacts with Bcl-xL, leading to the formation of heterocomplex and activation of the anti-apoptotic function [23]. HEMA was shown to be cytotoxic in a dose-dependent manner [24]. The mechanisms of HEMA-induced cell damage may be involved in the reduction of cellular glutathione and increased reactive oxygen species generation [15]. In addition, a previous study reported that HEMA can cause cell apoptosis through the intrinsic mitochondrial pathway by activating p53 [25]. The mechanisms behind the way in which fortilin protects cells from HEMA-induced apoptosis probably relate to its ability to bind to p53, which lead to inhibition of proapoptotic proteins [15]. Taken together, the full-length recombinant protein was chosen over other fragments to incorporate into the GIC specimens for the subsequent experiments. The amount of fortilin added into each specimen was 1 µg. This amount was based on a previous study that showed the protein released approximately 1% of the total protein during the early phase [26].

The biological properties of GICs modified with fortilin, TCP, or a combination of fortilin and TCP were investigated in terms of cell viability, cell differentiation, and mineral formation. The results of the MTT assay indicated that GIC and modified GICs were cytotoxic to hDPSCs during the first 3 days and the viable cells in all groups except GIC were increased on day 5. The cytotoxicity of the test materials during the early stage probably resulted from an acid–base reaction during material setting, components in the polyalkenoic acids, and fluoride release. However, cell viability in all groups except GIC was increased thereafter, indicating that the components in the modified GICs may help reduce the cytotoxic effect of conventional GIC. Dental pulp tissue contains various cell types including fibroblasts, and dental pulp stem cells/progenitor cells. In response to injury, preexisting odontoblasts or odontoblast-like cells are stimulated and subsequently form regenerative dentin. Dental pulp stem cells are multipotent stem cells, capable of differentiating into odontoblastic/osteogenic, chondrogenic, and adipogenic lineages [27]. Dentinogenesis and osteogenesis are very similar processes and share several common markers such as ALP and OPN [28]. ALP expression is upregulated during the early stage of odontoblastic differentiation, while DSPP and DMP-1, specific markers of odontogenic lineage [28,29], play important roles in dentin mineralization [30]. Our results revealed that in the GIC + TCP+ FL group, ALP, DSPP, and DMP-1 were upregulated during the process of mineralization, whereas the expression level of these genes was not markedly upregulated in the GIC+ FL and GIC + TCP groups. Furthermore, the result from the ALP activity assay showed higher levels of enzyme activity in the GIC + TCP+ FL group than those in the GIC+ FL and GIC + TCP groups.

These findings indicate that the combination of fortilin and TCP is likely to promote the odontogenic differentiation of hDPSCs better than fortilin or TCP alone. Among various bioactive compounds, TCP has gained much attention in biomaterials research due to its biocompatibility, osteoconductive, and osteoinductive properties. For dental applications, TCP has been utilized in several approaches such as material coatings, cements, and in combination with scaffolds or dental restorative materials [31,32]. TCP has been frequently used as a scaffold in tissue engineering, repair of bony defects, and promoting remineralization of carious lesions. It demonstrated good biocompatibility and osteoconductivity, thus enhancing bone growth on the surface [31]. A previous study showed its proliferative effect on osteoprecursors and dental follicle cells [33]. Furthermore, the TCP scaffold was shown to potentially induce odontogenic differentiation in dental pulp stem cells [34]. Dental pulp-derived stem cells cultured with TCP scaffold demonstrated upregulation of DSPP and DMP-1 expressions [34]. In contrast, our findings did not show upregulation of DSPP and DMP-1 in hDPSCs cultured with GIC modified with TCP. In the present study, GIC modified with a combination of fortilin and TCP or fortilin alone could enhance the calcium deposition in hDPSCs better than GIC supplemented with TCP. Generally, the ability of calcium phosphate compound in mineralization promotion largely depends on the content of calcium and phosphate in the microenvironment. In this study, the calcium phosphate amount in GIC modified with TCP may be insufficient to promote either odontoblastic differentiation or mineral deposition. Alternatively, the calcium phosphate form incorporated in the GIC after setting may not be readily released from the bulk material. Altogether, the combination of fortilin and TCP appeared to have better beneficial effects than fortilin or TCP alone. The mechanistic explanation of this combinatorial effect remains to be elucidated.

The major limitation of this study was that this in vitro model cannot fully simulate the complexity of multicellular networks and the cell–biomaterial interactions. In the present study, hDPSCs were in close contact with the test material, which may differ from the clinical scenario. In a deep carious lesion, GIC can be used as a liner and placed on the thin layer of remaining dentin. The active components of the GIC may diffuse through the dentinal tubules and exert their effects on the underlying cells. It would be of interest to investigate the effect of this modified GIC on cell migration in a transwell model.

## 5. Conclusions

Full-length fortilin is the most suitable recombinant protein that can enhance cell viability and increase cell survival from HEMA-induced cytotoxicity. GIC incorporated with fortilin and TCP induces odontogenic differentiation and mineral deposition in hDPSCs. Further study is warranted concerning the physical properties and in vivo biocompatibility of this modified GIC.

## Figures and Tables

**Table 1:** List of materials investigated in this study.

Groups	Powder Components
GIC	100% calcium fluoroaluminosilicate glass and 5% copolymer acid
GIC + TCP	99.95% calcium fluoroaluminosilicate glass, 5% copolymer acid, and 0.05% tricalcium phosphate
GIC + FL	100% calcium fluoroaluminosilicate glass, 5% copolymer acid, and 1 µg fortilin
GIC + TCP + FL	99.95% calcium fluoroaluminosilicate glass, 5% copolymer acid, 0.05% tricalcium phosphate, and 1 µg fortilin

**Table 2:** The primer sequences (5′–3′) used for qRT-PCR *.

Gene	Primer (5′–3′)	GenBank Accession No.
ALP	F: CCACAAGCCCGTGACAGA	NM_001127501
	R: GCGGCAGACTTTGGTTTC	
OPN	F: ACACATATTGATGGCCGAAGGTGA	NM_00582.2
	R: TGTGAGGTGATGTCCTCGTCTGT	
DMP-1	F: GCAGAGTGATGACCCAGAG	NM_004407.3
	R: GCTCGCTTCTGTCATCTTCC	
DSPP	F: GGGATGTTGGCGATGCA	NM_014208.3
	R: CCAGCTACTTGAGGTCCATCTTC	
GAPDH	F: GCACCGTCAAGGCTGAGAAC	NM_001289745.1
	R: ATGGTGGTGAAGACGCCAGT	

* Abbreviations: ALP, alkaline phosphatase; OPN, osteopontin; DMP1, dentin matrix protein 1; DSPP, dentin sialophosphoprotein; GAPDH, Glyceraldehyde-3-phosphate dehydrogenase; F, forward; R, reverse.

## Data Availability

Not applicable.
